# Metabolomics in Bone Research

**DOI:** 10.3390/metabo11070434

**Published:** 2021-07-01

**Authors:** Jingzhi Fan, Vahid Jahed, Kristaps Klavins

**Affiliations:** 1 Rudolfs Cimdins Riga Biomaterials Innovations and Development Centre of RTU, Institute of General Chemical Engineering, Faculty of Materials Science and Applied Chemistry, Riga Technical University, Pulka St 3, LV-1007 Riga, Latvia; jingzhi.fan@rtu.lv (J.F.); vahid.jahedzomorrodi@rtu.lv (V.J.); 2 Baltic Biomaterials Centre of Excellence, Headquarters at Riga Technical University, Pulka St 3, LV-1007 Riga, Latvia

**Keywords:** metabolomics, bone homeostasis, osteoporosis, bone regeneration, osteosarcoma, biomaterials

## Abstract

Identifying the changes in endogenous metabolites in response to intrinsic and extrinsic factors has excellent potential to obtain an understanding of cells, biofluids, tissues, or organisms’ functions and interactions with the environment. The advantages provided by the metabolomics strategy have promoted studies in bone research fields, including an understanding of bone cell behaviors, diagnosis and prognosis of diseases, and the development of treatment methods such as implanted biomaterials. This review article summarizes the metabolism changes during osteogenesis, osteoclastogenesis, and immunoregulation in hard tissue. The second section of this review is dedicated to describing and discussing metabolite changes in the most relevant bone diseases: osteoporosis, bone injuries, rheumatoid arthritis, and osteosarcoma. We consolidated the most recent finding of the metabolites and metabolite pathways affected by various bone disorders. This collection can serve as a basis for future metabolomics-driven bone research studies to select the most relevant metabolites and metabolic pathways. Additionally, we summarize recent metabolic studies on metabolomics for the development of bone disease treatment including biomaterials for bone engineering. With this article, we aim to provide a comprehensive summary of metabolomics in bone research, which can be helpful for interdisciplinary researchers, including material engineers, biologists, and clinicians.

## 1. Introduction

Bone is the primary tissue in the skeleton system. At first glance, bone tissue might appear to be a static and rugged part of the body with almost no dynamics. However, the first impression is deceiving. Bone homeostasis is a very dynamic and complex process closely linked to other organism functions, including immunity. Moreover, the disturbances in these processes are associated with numerous diseases, chronic or acute, that severely affect life quality and are even life-threatening. 

Among them, osteoporosis, the most well-known bone disorder, has a high prevalence rate, and the rate for osteopenia is even higher [1]. This chronic bone disease has a preference among postmenopausal women (over 10%) and the aged population [2,3], and it highly increases the risk of osteoporotic fracture. Pathologic fracture can also arise from weakened bone caused by tumors. Bone fracture, including traumatic and pathological caused, is a malignant medical condition that frequently happens for various reasons. The loss of productivity and individual disability after fracture dictates a high demand for hard tissue healing studies. It has become evident that a much deeper understanding of hard tissue and related diseases is needed to develop a novel diagnosis, prognosis, and treatment approaches. The need for this will only increase due to the ageing population.

In this regard, metabolomics—a large-scale study of metabolites that are directly involved in biochemical activity holds great promise to advance our knowledge on bone tissue (patho)physiology. Describing the metabolite pathways involved in a disease can greatly promote understanding disease development, diagnosis, and prognosis [4]. Moreover, the prominent biomarker identification can be incorporated with the traditional diagnostic methods to improve diagnostic accuracy. Additionally, metabolite ability to modulate phenotype directly through biochemical reaction can pave the road for developing new treatment approaches, especially blockers, competitive inhibitors, and accelerants. 

**Figure 1:**
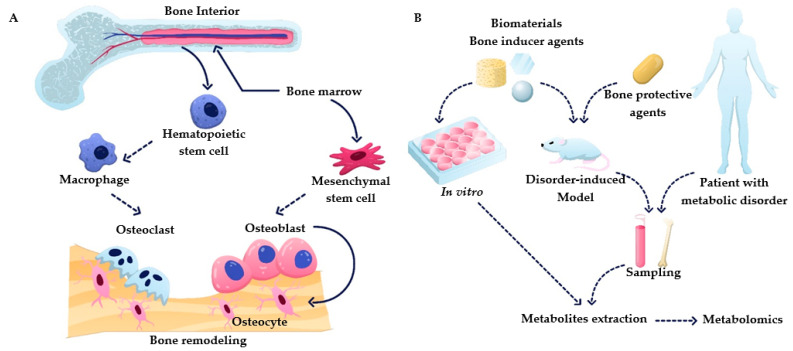
(**A**) Overview of bone remodeling processes, including osteogenesis and osteoclastogenesis. (**B**) Summary of metabolomics applications in bone research.

In recent years, there has been a growing interest in metabolomics for bone tissue-related research, which has resulted in an improved understanding of underlying cellular processes during bone homeostasis and disease. However, the available information about bone–immune system and cell–cell interactions is still sparse and systematic studies are needed to deepen our understanding about these interactions. This review provides an overview of the metabolic processes relevant to bone homeostasis and the most prevalent bone diseases. It summarizes recent studies that employ metabolomics to better understand bone metabolism (Figure 1B). With this review, we want to showcase the role and value of metabolomics in bone research and encourage incorporating metabolomics in more bone-related studies, as it is providing unique insights. 

## 2. Metabolism of Bone Cells

### 2.1. Bone Homeostasis

Bone is the primary tissue in the skeleton system, providing the function of structure support, hematopoiesis location, and mineral storage. To perform these functions, bone has a complex structure that contains compact bone and cancellous bone, with the composition of organic and inorganic composites containing living cells [5]. The bone remodeling process is a cascade of regular activities in which three cell types, including osteoblasts, osteoclasts, and osteocytes, play crucial roles (shown in Figure 1). Bone undergoes continuous resorption carried out by osteoclasts and formation by osteoblasts, which are linked cell activities that form a dynamic equilibrium in bone tissue [6]. To briefly summarize this complex process, osteoclast precursors are activated to start resorption stages by cytokines such as receptor activator of nuclear factor-kB (RANK) and macrophage colony-stimulating factor (M-CSF) released by bone lining cells upon receiving a biochemical signal such as hormones (e.g., parathyroid) or mechanical signal (due to bone damage). Osteoclasts, specialized cells developed from the monocyte/macrophage lineage, can contact the bone matrix to form a sealed bone resorption area, playing a role in degrading bone tissue [7]. The activity of the osteoclast is terminated by the formation of resorption pits on the bone surface. During the formation process, osteoblasts deposit the osteoid into the resorbed site to form a mineralized matrix. Osteoblasts are then either apoptotic or buried as osteocytes in new bone layers. It is crucial to maintain a balance between osteoclasts and osteoblasts. Bone remodeling is a dynamic and lifelong process. An imbalance in this process leads to bone disorders. It can cause several diseases, such as overactive osteoclasts and bone turnover, which would lead to osteopenia and osteoporosis, while abnormal osteoblasts elevate the risks for hyperosteogeny and tumor [3,8].

Cell differentiation is an integral part of bone homeostasis, as the osteoclast is derived from hematopoietic cells, while osteoblasts are produced from the differentiation of bone marrow stem cells (MSCs). The differentiation from MSC to osteoblast and from monocyte-macrophage lineage to osteoclast involves metabolic changes and adaptions. Metabolomics is one of the key methodologies to study these processes and deepen the understanding of osteogenesis and osteoclastogenesis and their relationship with bone diseases. MSCs, the precursor of osteoblasts, predominantly utilize glycolysis as the energy source within the bone marrow; however, the energy metabolism shifts to oxidative phosphorylation during the differentiation [9,10]. Hypoxia-inducible factor 1 α (HIF-1α) works as a regulator of MSC energy metabolism during osteogenic and chondrogenic differentiation [11]. HIF-1α can promote glycolysis and inhibit oxidative phosphorylation (oxidative phosphorylation (OxPhos)) and is most active in undifferentiated MSCs [12]. MSCs have been shown to grow and proliferate in the glycolysis pathway in hypoxic condition. At the same time, they undergo a metabolic shift to the tricarboxylic acid (TCA) pathway to supply the required energy to differentiate into the bone cells [9]. As a result, a higher OxPhos activity with an unaltered or decrease in glycolysis happens in MSCs differentiation [9,12,13]. In mature osteoblasts, glycolysis produces most of the needed energy for osteoblasts to maintain the functionality [14]. The well-known osteogenic factor, bone morphogenetic protein, can boost glucose metabolism and therefore enhance the development of bone and cartilage [15].

During osteoclastogenesis, both glycolysis and OxPhos were found increased, as indicated with a higher glucose consumption and lactate production [16]. This means that glycolysis and mitochondrial processing both play a role in the differentiation and absorption. Increased expression of glycolytic genes, including hexokinase, phosphofructokinase, and pyruvate kinase, was found during osteoclastogenesis [17]. The final osteoclasts may become apoptosed, which makes them difficult to study [18]. However, there is some evidence which indicates an alternative fate for RANKL-stimulated osteoclasts-osteomorphs production, [19]. The lifespan of osteoclasts is generally considered to be as long as 2 weeks [20]. Nevertheless, Jacome-Galarza et al. described a mechanism upon which osteoclasts could be maintained in bone, leading to compensate for osteoclast deficiency in vivo [21]. 

Lipid metabolism can also alter the differentiation and function of bone cells. Long-chain polyunsaturated fatty acids, such as docosahexaenoic acid and arachidonic acid, can directly suppress the differentiation and activity of osteoclasts [22]. As part of lipid rafts to control the signal transduction during osteoclastogenesis, cholesterol can alter the RANK-RANKL signal transduction, resulting in increased bone resorption under a high content of cholesterol [23]. Similarly, phosphoinositide, a membrane lipid, regulates calcium signaling and influences osteoclast differentiation [23]. The changes of 18 metabolites, most of which were lysophosphatidylcholines, were detected in the study of differentiation inhibition with estradiol [24]. The enzymatic expression changes explained this inhibition during osteoclast differentiation and oxidative/anti-oxidative imbalance during osteoclast proliferation. It also should be pointed out that lipids are used as an energy source which in turn can influence cell functions. Low lipid levels in the skeletal stem cells environment leads to chondrogenesis rather than osteogenesis due to the osteoblasts dependency on the fatty acid oxidation (fatty acid Β-oxidation (FAO)) [25].

The metabolism studies on osteogenesis and osteoclastogenesis provide a guide to regulate the differentiation of precursor cells by altering the metabolic pathways. Monitoring the cell function upon exposure to a change in its surrounding environment allows for controlling cells’ behavior. Osteogenesis differentiation from MSCs can be guided by parathyroid hormone by enhancing glycolysis to increase bone formation [24]. Lee et al. attempted to regulate adipocyte and osteoblast differentiation from MSCs using several metabolites. Among them, ergosterol peroxide and 9,11-dehydroergosterol peroxide were found to inhibit the differentiation toward adipocytes. Diterpenes dehydroabietic acid, 7-oxocallitrisic acid, and pimaric acid, on the other hand, induced osteoblasts differentiation [24]. The effect of high and low amounts of energy source, oxygen, and ROS as environmental factors on a deviation of cellular behavior has been well reviewed recently [6]. 

### 2.2. Osteoimmunology

The close connection between the immune system and bone tissue generated the conception of osteoimmunology [26]. Immune cells have a remarkable effect on bone physiology by acting on osteogenesis and osteoclastogenesis. The activity of bone resorption is also regulated by interleukins, cytokines secreted by immune cells. Among all immune cells, macrophages play an essential regulatory role in bone regeneration. Macrophages are divided into two polarized extremes, pro-inflammatory M1 and anti-inflammatory M2 [27]. Extracellular amino acid levels can alter the immune system functions through altering macrophage phenotypes. Arginine (Arg), ornithine, tryptophan, and glutamine can be considered amino acid-based immunomodulators [28]. Arginine metabolism plays a critical role in macrophage polarization and characterization. The prominent metabolic feature of M1 macrophages is Arg catabolism to nitric oxide (NO) and citrulline using nitric oxide synthase (NOS). While for M2 macrophages, Arg is catabolized to ornithine and urea through arginase [27]. Therefore, the catabolic and anabolic activity of NOS and arginase processes can regulate the polarization of macrophage and thus influence the inflammation and regeneration in hard tissue [29]. Indeed, the chronic inflammation of the target tissue can be pharmacologically controlled by regulating NO and Arg production [30]. 

In bone regeneration processes, activation of M1 macrophages in the early inflammatory phase leads to the secretion of inflammatory cytokines such as tumor necrosis factor α (TNF-α), Interleukin 6 (IL-6), and IL-1b, which results in activation of the osteoclastogenesis cascade with consequential bone resorption [31]. The M1 macrophage effect on mesenchymal stem cell (MSC) osteogenesis through the COX-2-prostaglandin E2 pathway has also been reported [32]. M2 macrophages are involved in the secondary stages of bone repair, which can lead to fibrous capsule or bone formation. Fibrous capsule formation prevents bone marrow stem cells being connected to the surface of a biomaterial for further progress in osteogenesis. Therefore, the domination of the inflammatory phase of macrophages guarantees the long-term failure or success of bone regeneration in the subsequent stages. The protracted stage of the M1 phenotype results in more fibrous inducible cytokines are amplified and fibrous capsules are formed, helping M2 phenotypes [31]. The immune response elicited by primary M1 macrophages in the early stages determines the decision of secondary M2 macrophages to secrete specific cytokines. In summary, macrophages positively contribute to bone healing by secretion of osteogenesis-related growth factors or negatively interfering through fibrous-induced inflammatory cytokines. 

From an energy metabolism point of view, macrophages are constantly switching from glycolysis to oxidative phosphorylation (OxPhos). It was proved that M1 phenotypes use glycolysis and pentose phosphate pathways to uptake required ATP. M1 phenotypes lead to metabolic perturbations in several metabolites of the TCA cycle, including citrate, succinate, and itaconate. Prolonged M1 polarization can be selected as a metabolic marker for the diagnosis of inflammatory diseases such as rheumatoid arthritis and metabolic disorders such as diabetes and osteoporosis [28]. On the contrary, glycolysis has been shown to not be relevant for M2 polarization, while the consumption of glutamine Gln can be upregulated to fuel the TCA cycle [33]. Elevated FAO and OxPhos pathways indicate M2 macrophage activity and sustain the inflammatory response [27]. Gln metabolism has been reported as the synergistic support for macrophage activation through M2 phenotype differentiation [34]. Macrophages have the potential to uptake different types of lipids, including very low-density lipoprotein (VLD), low-density lipoprotein (LDL), and oxidized lipoproteins. Lysosomes convert the consumed lipids to free fatty acids and cholesterol, which eventually are involved in OxPhos and ETC pathways. As lipid-based macrophage modulators, fatty acid synthesis (FAS) and FAO guide M1 and M2 macrophage polarization, respectively [28]. This suggests that the tracking of metabolites involved in macrophage energy homeostasis can serve as a marker to evaluate a transition from the M1 phenotype towards M2. In fact, it has been demonstrated that metabolomics, through an elucidation of changes in macrophage polarization, provides accurate information about an inflammatory disease [28,35].

## 3. Metabolomics in Research of Bone Diseases

As demonstrated in the previous section, metabolism plays a crucial role in bone homeostasis, and disturbances can cause or are caused by several diseases. Therefore, it is evident that metabolite measurements could provide a valuable readout for bone disease research. Indeed, metabolomics has been used for the identification of possible biomarkers for the diagnosis and prediction of various bone-related diseases. Besides diagnosis, metabolomics has also been applied to monitor the treatment. Perturbations in several major metabolic pathways due to bone diseases have been reported. Specifically, arginine and its related metabolism pathways are critical in osteoimmunology. Energy metabolisms, including glycolysis and TCA cycle, are pivotal for bone cell differentiation and function. polyunsaturated fatty acids (PUFAs) are valuable for dietetic, clinical, and biological research of osteoporosis and arthritis. Furthermore, fatty acids, such as arachidonic acid, are exploitable to develop advanced treatments. The metabolites related to acute and chronic inflammation are also closely involved in bone defects. Additionally, energy-related metabolites such as lactate and glutamine are altered in bone cancer. These metabolites and pathways can be utilized to develop diagnosis, prognosis, and treatment procedures of bone diseases.

### 3.1. Osteoporosis

Osteoporosis (OP) is the most known and common bone disease. OP occurs worldwide in all populations, with a higher prevalence among postmenopausal women and aged people. A low bone mineral density, which increases fracture risk, is the hallmark of OP. OP is usually characterized by metabolic disorders of bone tissue, and usage of metabolomics to study pathophysiology has gained popularity [36]. Animal models are commonly used to study the molecular mechanisms of OP in vivo. Ovariectomized (OVX) mice is an animal model of OP that mimics postmenopausal women with low BMD. Using this model, ovarian extraction results in a decrease in estrogen and progesterone, with consequences elevating the rate of bone loss [36]. Lipid metabolisms, especially arachidonic acid metabolism, linoleic acid metabolism, and glycerophospholipid metabolism, are impacted by the decrease in estrogen [37,38]. Polyunsaturated fatty acids (PUFAs) are well known for their influence on BMD [39]. Two different families of PUFAs, n-3 PUFA, derived from α- linolenic acid, and n-6 PUFA, derived from linoleic acid, are frequently occurring in metabolomics studies with their opponent functions [22]. In fact, elevated levels of arachidonic acid (AA), an n-6 PUFA, have been observed in oophorectomized rats and postmenopausal women with OP [37,38,40]. Arachidonic acid can stimulate the expression of receptor activator of NF-kB ligand (RANKL), leading to a high plasma RANKL level [22]. As the cytokine essential for osteoclast differentiation, a high concentration of RANKL can remarkably promote osteoclastogenesis, resulting in the loss of bone tissue [41]. On the contrary, docosahexaenoic acid (DHA), an n-3 PUFA, suppresses osteoclast formation from human CD14-positive monocytes by the reduction of key signaling transduction pathways of kinases (JNK, ERK, and p38 MAPKs) [22,42]. Thus, DHA inhibits osteoclastogenesis by blocking RANKL-induced activation from primary macrophages. At the same time, prostaglandin and leukotrienes, the downstream metabolic products of arachidonic acid, were also proved to impact Wnt signaling (osteoblastogenesis) and osteoclastic resorption, respectively [43,44]. A high-level accumulation of other lipid metabolites has been found in postmenopausal women and oophorectomized rats as well [45,46,47]. Lipid metabolisms not only impact osteoclastogenesis but also influence osteoblastogenesis. The increased lipid oxidation, followed by high oxidative stress, can activate peroxisome proliferator-activated receptor γ, consequently inhibiting osteoblastogenesis and promoting adipogenesis [48]. PUFAs also play various roles in osteoblastogenesis. Fatty acids can activate peroxisome proliferator-activated receptor γ (PPARγ) [49]. PPARγ plays a pivotal part in cell-fate determination, guiding the MSCs to differentiate into adipocytes [50]. This could explain the bone loss of the patients with a high-fat diet. AA decreases the expression of osteogenic markers and the osteoprotegerin/RANKL ratio, causing the appearance of adipocytes in MSC differentiating during osteoblastogenesis [51]. However, n-3 PUFA such as DHA and eicosapentaenoic acid do not have such an impact on MSCs [51,52]. In an attempt to characterize the pathological mechanism of the postmenopausal OP, metabolomics was employed to analyze the OVX mice-related femur tissue. The obtained data indicated altered levels of 93 lipid metabolites such as fatty acyls, glycerolipids, glycerophospholipids, sphingolipids, and sterols, among which levels of many fatty acids were increased in the OVX model [53]. To sum up, PUFAs play a critical role in osteoporosis by (1) promoting osteoclastogenesis through the expression of RANKL and (2) altering the differentiation of MSCs by inducing adipogenesis. 

Ageing is another leading cause of low BMD. The fractures due to senile OP are highly life-threatening for the aged population, especially those over the age of 70 [1]. It is known that the functionality of osteoblasts, adipocytes, and osteoclasts changes with ageing. A higher adipogenesis level was estimated with the accumulation of bone marrow fat, not found in postmenopausal OP, which may be a critical cause for lower osteoclastogenic activity [54,55]. An in vitro study verified that adipocytes, with their metabolites, inhibit osteoblast differentiation by downregulating histone acetylation [56]. Again, lipid metabolism appears to have an essential role in the age-associated reduction of BMD, as demonstrated by a metabolomics study on senescent osteoblasts [57]. The authors identified the downregulation of n-3 PUFAs in the fatty acid metabolism due to the severe oxidative stress damage. The decrease in n-3 PUFAs, as explained before, could result in an elevation of bone loss through osteoclasts. 

At the clinical level, a plasma metabolites profiling conducted on 1552 individuals to identify BMD-associated metabolomic markers detected a higher level of creatine, dimethylglycine, and glycine [58]. The authors suggested that these metabolites were causally negatively associated with BMD by altering bioenergetic processes as well as glycine and threonine metabolism pathways. As a major component of the protein collagen, hydroxyproline is also considered an OP-specific marker [59]. Serum metabolite components of postmenopausal women with low BMD were investigated using CE-MS and showed that the level of hydroxyproline could be a marker of OP [60]. Increased concentration of hydroxyproline indicates the degradation of collagen type I from the bone matrix and it was reduced after treatments [61]. Similarly, other catabolites developed in bone from collagen, such as deoxypyridinoline (collagen stabilizer) and pyridinoline (cross-linking compound of collagen fibers), can also serve as the markers of OP [59]. However, changes in their levels are the results rather than the causes of OP; therefore, they are more suitable for the development of diagnosis but not treatment.

### 3.2. Bone Injuries

Bone fractures have always been a relevant threat to public health worldwide. Several studies have recently been published investigating the metabolic processes during both inflammatory response and tissue regeneration after bone fractures. 

Ibrahim et al. identified the changes in the plasma metabolite levels in patients who went through intramedullary reaming [62]. The precursors to extracellular matrix proteins, lipids, and cysteine displayed remarkable elevation after the surgery. At the same time, the abundance of tryptophan decreased. Galactosamine and glucuronic acid, as the precursors of chondroitin sulphate, were elevated in the after-reaming plasma. The precursors of hyaluronic acid, such as acetylglucosamine, were also increased. The increase in these ECM building blocks indicates that the matrix remodeling is accelerated after the surgery. The increased lipids, including ceramides, myristoleate, and phosphoethanolamine, play well-recognized roles in inflammation. They modify inflammatory processes by impacting inflammatory cell signaling and gene expression patterns [63]. Additionally, glutathione and taurine, the downstream products of cysteine, are both antioxidants, protecting the cells from reactive oxygen species [64,65]. At the same time, 4-hydroxynonenal, which contributes to oxidative stress, was reduced after the reaming [66]. Linolenate, a precursor of arachidonate (an inflammatory mediator), was found to be increased, much like corticosterone and cortisol, which are inhibitors in the proinflammatory pathway [67]. Kynurenine, a metabolite of tryptophan, was elevated in the post-reaming plasma, while tryptophan itself declined, pointing towards an upregulated activity of the kynurenine pathway [68,69]. It should be pointed out that the increase in the kynurenine/tryptophan ratio has been considered a marker for indoleamine 2,3-dioxygenase (IDO) activity [69]. Thus, based on the anti-inflammatory mechanism of IDO, the increase in kynurenine benefits anti-inflammatory processes as well [70]. Due to the anti-inflammatory function, IDO is considered a critical regulator in graft-versus-host disease, and as such the mediation of IDO is important for implantable biomaterials [71]. Additionally, kynurenic acid, a metabolite of kynurenine through transferase, decreased the release of TNF-α, further reducing inflammation response [69,72]. Taking it all together, the metabolites profiling after surgery indicated that the body tends to reduce the inflammation response after wound healing. 

Bone turnover markers, including the biomarkers of bone resorption, bone formation, and osteoclast regulatory proteins, have been utilized to evaluate bone homeostasis. As a result, the measurement of these markers can be employed to monitor the healing of fractured bones [73]. Veitch et al. detected the alterations of these markers following tibial shaft fracture in 24 weeks [74]. Both bone resorption and formation markers were elevated, which was in line with the observation from the animal model study using sheep conducted by Sousa et al. [75]. In the first two weeks, the catabolites of type-I collagen-like C-terminal telopeptide (bone resorption marker) had a higher increase compared to the bone formation markers, such as bone alkaline phosphatase and osteocalcin [74]. This highlighted that the activity of necrotic tissue resorption is high at the beginning stage of wound healing, following the reconstruction of bone tissue.

The metabolic signatures provide valuable clues to establish “the big picture” on bone regeneration from critical injury. There are seemingly microscopic but significant differences in defect and injury types; however, only a few studies are available. Therefore, further metabolite profiling studies considering all aspects of bone injuries are needed to understand the molecular mechanisms affected by injury and healing. 

### 3.3. Rheumatoid Arthritis

Rheumatoid arthritis (RA) is commonly considered arthritis driven by autoimmune pathogenesis. The analysis of joint tissue metabolism, especially with immune cells, can ameliorate the understanding of pathogenesis and prognosis. The bone destruction of rheumatoid arthritis happens mainly due to the abnormal activation of osteoclasts [76]. Therefore, the overactivity of osteoclasts stimulated by various factors may cause and/or aggravate arthritis. Similar to the PUFAs in osteoporosis, n-6 PUFAs, as the precursor of inflammatory eicosanoids, are responsible for the cartilage loss and inflammation in RA [77]. The influence of n-3/n-6 PUFA intake on RA has already been extensively studied among dietary studies and clinical medicine, which suggest increasing the intake of n-3 PUFAs such as DHA and to avoid the intake of n-6 PUFAs such as AA [63,78,79]. Another fatty acid family, short-chain fatty acid (SCFA), is also relevant for arthritis. Lower butyrate (SCFA) levels in the blood of RA patients and arthritic mice have been reported [80]. The amelioration of the severity of systemic autoimmune inflammatory conditions was achieved by oral administration of SCFAs [81]. Furthermore, the butyrate ability to suppress the expression of inflammatory cytokines from T cells by promoting the expression of IL-10 has been demonstrated [82]. 

Energy metabolism is also abnormal in RA. Narasimhan et al. performed NMR-based metabolite profiling of the serum from patients with rheumatoid arthritis to identify the synovial joint biomarkers [83]. The metabolites with abnormal levels found in RA serum were associated with the TCA cycles, fatty acid, and amino acid metabolism. Among them, lactate and pyruvate were significantly upregulated. This can be explained by the higher bioenergetic and biosynthetic demands in the inflamed tissue [83]. The RA metabolic process of T cells that drives tissue inflammation could be attributed to the mitochondrial defect [84]. The mitochondrial DNA containments trigger T cell tissue invasion in the organelle assembled inflammasome and caspase-1. Another study demonstrated that the sugar metabolism of fibroblast-like synoviocytes was significantly disturbed by RA [85]. Kim et al. also pointed out that the sugar metabolism level in RA is higher than healthy people and higher than osteoarthritis, which makes it a distinct feature for RA [85]. As presented in the previous chapter, a high glycolysis level is a characteristic of osteoclastogenesis from monocytic progenitors with an increased glycolytic genes expression [17]. This switch of energy consumption can explain the tendency towards glycolysis and mitochondrial defect in RA. The symptoms of arthritis are caused by the destroyed cartilage; consequently, the abnormal enhancement of osteoclast and/or immune cell activity by fatty acids or glycolysis level is closely related with the disease conditions.

### 3.4. Osteosarcoma

As the most common histological form of the bone cancerous tumor, osteosarcoma causes severe symptoms by malignant neoplasia, threatening people of all ages [86,87]. A clear presentation of metabolic heterogeneity in oncogenesis and metastasis benefits early diagnosis and enhances the understanding of the clinical behavior of tumor tissue. Among all the pathologies, osteoblastic tumor holds the highest proportion, followed by chondroblastic and fibroblastic [86,88]. Several studies have been performed on osteosarcoma with a focus on abnormal metabolisms. An NMR profiling on blood serum samples revealed that energy metabolism was enormously altered throughout tumorigenesis [86]. The abnormal levels of amino acids, fatty acids, and glucose were found, suggesting an alternative energy source for cancer cells. In most cancer cases, glycolysis is considered to be the primary energy source for tumor tissue, while oxidative phosphorylation level is decreased [89]. A similar decrease in TCA cycle metabolite levels was detected in an in vitro experiment using osteosarcoma stem cells [90]. This preference for energic metabolism was verified by metabolite enrichment analysis, of which metabolite biomarkers involved in the glycolysis pathway were highly evaluated [90,91]. The lower TCA cycle level in osteosarcoma tumor tissue was explained by the down-regulation of mitochondrial function, accompanied by a reduction of glutamate, aspartate, and glutathione with an elevation of Gln [90,92]. The mitochondrial dysfunction caused by the expression of metastasis genes in osteosarcoma cells resulted in metabolic disorder and presented an aggressive phenotype [93]. 

The metabolite-based osteosarcoma biomarkers can be potentially employed not only for diagnosis but also to monitor disease progression. These metabolites can be attributed to the development of regulators for energy metabolism and cellular stress in osteosarcoma tumor tissue. Besides, targeting metabolic pathways provides new therapeutic strategies. For example, new osteosarcoma therapy targeting the amino acid metabolism of cancer stem cells has been suggested [90].

### 3.5. Summary of Metabolic Pathways Relevant in Bone Diseases

To comprehensively identify metabolic pathways that are affected during bones diseases, we conducted an integrating enrichment and pathway topology analysis from the data of Table 1 by using Metabo Analyst 5.0 [94]. For this, we used the metabolites that are reported as significantly altered in osteoporosis, arthritis, and osteosarcoma.

The metabolic pathways prominently altered in both osteoporosis and arthritis are (1) aminoacyl-tRNA biosynthesis, (2) arginine and proline metabolism, (3) arginine biosynthesis, and (4) valine, leucine and isoleucine biosynthesis. At the same time, the most altered pathways from osteosarcoma are (1) alanine, aspartate and glutamate metabolism, (2) TCA cycle, and (3) arginine biosynthesis. Interestingly, a high degree of similarity of the most altered metabolic pathways has been found between osteoporosis and arthritis. This phenomenon may be caused by the pathophysiological similarity of these two diseases: abnormal resorption of the target tissue by osteoclasts, macrophages and/or some other immune cells. However, due to the limitation of the data sources and the diversity of various original studies, this conclusion needs further research and discussion. Most notably, arginine synthesis and metabolism are remarkably altered in all mentioned diseases. The metabolism of glutamate, a precursor of arginine, is also involved in OS. The immune system, therefore, plays a critical role in bone diseases as a regulator of immune responses and arginine, and its related metabolic pathways have great value for the future bone research [95]. It should be pointed out that metabolic pathways altered in bone diseases have been previously reported to be relevant in other diseases. The control of physiopathological processes such as angiogenesis, inflammation, and tumorigenesis is related to aminoacyl-tRNA synthesis [96]. Arginine and proline have important roles in wound healing, antioxidative reactions, and immune responses, as a result, the metabolites’ variation after tissue injury is rational [97]. Obesity and cancer risk are also linked with proline metabolism, leading to various of complications [98]. The alteration of glycolysis, TCA cycle and glutaminolysis are typical signals in cancers, [99,100]. To conclude, some of the changes in metabolism observed in bone diseases reflect more general pathophysiological processes such as inflammation. Further studies are needed to elucidate how bone diseases may influence or be influenced by other diseases under a certain physical condition of the patients.

**Table 1:** Overview of reported metabolite changes in the different diseases, used analytical methods and corresponding references.

Ref.	Disease	Technique	Sample Type	Metabolite Changes
[46]	POP	GC/TOF-MS	Rat plasma	↑ Arachidonic acid, octadecadienoic acid, valine, leucine, isoleucine, homocysteine, hydroxyproline, ketone bodies ↓ Docosahexaenoic acid, dodecanoic acid, lysine
[53]	POP	UPLC/Q-TOF-MS	Rat bone tissue	↑ Lysophosphatidylcholine, phosphatidylcholine, ceramide, phosphoserine ↓ Uridine, hypoxanthine, xanthine, inosine, cytidine, phenylalanine, leucine, carnitine, proline, arginine
[101]	POP	1H NMR	Rat urine	↑ Trigonelline, phosphocreatine, pyruvate, methylamine, trimethylamine oxide ↓ Benzoic acid, dimethylamine, trimethylamine, threonic acid, alanine, leucine, 2-ketoglutarate, allantoin, acetate, formate
[47]	POP	GC/TOF-MS	Rat plasma	↑ Arachidonic acid, homocysteine, homocysteine, ethanedioic acid↓ Alanine, malic acid, citric acid, fructose involved
[45]	POP	GC-MS	Women serum	↑ Arachidonic acid, lysine, eicosadienoic acid, oleic acid, linoleic acid, allose, tryptophan ↓ Homoserine, 3-hydroxy-l-proline, pyruvic acid
[102]	POP	UPLC-Q-TOF/MS	Rat serum	↑ Lysine, linoleic acid, hippuric acid, octadecadienoic acid, carnitine, glucose, arginine, S-adenosylhomocysteine, ornithine, tryptophan, arachidonic acid, methionyl-hydroxyproline ↓ Homoserine, 3-hydroxy-l-proline, pyruvic acid, citric acid, estriol, 8-HETE, uric acid, glutamine, glyceraldehyde, palmitic acid, 4-oxoretinol, taurocholic acid
[103]	DOP	NMR	Human plasma	↑ Leucine, isoleucine valine, alanine, N-acetylglycoprotein, inositol, proline, glucose, glutamine, 1-methyl-histidine, tyrosine ↓ O-acetylglycoprotein, α-ketoglutaric acid, citrate, creatine
[83]	RA	NMR	Human synovial	↑ Threonine, xanthine, methylsuccinate, glutamate, methylmalonate, taurine, lactate, pyruvate, propylene glycol, leucine, tyrosine, 3-hydroxybutyrate ↓ Creatinine, creatine, o-acetyl carnitine, L-carnitine, betaine, choline, formate, glycine, asparagine, formate, acetate, phenylalanine, succinate, pantothenate, fumarate, acetoacetate, acetone, lysine
[85]	RA	GC/TOF-MS	Human synoviocytes	↑ Inosine, urate, guanine, behenic acid, palmitoleic acid, arachidic acid, oleic acid, glucose-6-phosphate, phosphogluconic acid, aspartate, adipate, asparagine ↓ Isoleucine, leucine, leucine, histidine, valine, ornithine, lysine, methionine sulfoxide, tryptophan, mannitol, xylose
[104]	RA	LCMS	Human plasma	↑ Kynurenine, indolelactic acid, hypoxanthine, cholesterol, triglyceride, lysophospatidylcholines ↓ Tryptophan, fatty acids, acylcarnitines
[105]	OA	MALDI-MSI	Human bone marrow MSCs	↑ Arachidonic acid, oleic acid, stearic acid, dihydroxyacetone phosphate, phosphatidylglycerol, phosphatidylinositol ↓ Myoinositol, phosphatidic acid, lysophosphatidic acid, glutamine
[90]	OS	UHPLC-QE-MS	Human osteosarcoma stem cells	↑ Aspartic acid, asparagine, glutamine, arginine, ornithine, methionine, methylthioadenosine ↓ succinic acid, citric acid, aconitic acid, oxoglutaric acid, ureidosuccinic acid
[106]	OS	UHPLC-HRMS	Human serum	↑ Adenosine monophosphate, inosinic acid, guanosine monophosphate, hypoxanthine, lactic acid ↓ Uric acid, 4-hydroxybenzoic acid, testosterone sulfate, iminodiacetic acid, 3-carboxy-4-methyl-5-propyl-2-furanpropionic acid, decanoylcarnitine
[107]	OS	GC-MS	Human serum and urine	↑ Cystine, 2-hydroxybutyrate, inosine, creatinine, putrescine, aspartate, proline, galactopyranose ↓ Malate, fumarate, pyruvate, lactate, sucrose, dodecanoate, glycerol phosphate, creatinine
[62]	Bone Injuries	UPLC-MS/MS	Trauma patient plasma	↑ Myristoleate, hexadecadienoate, octadecadienedioate, choline phosphate, phosphoethanolamine, pregnenolone sulfate, cortisol, glycerol 3-phosphate, beta-citrylglutamate, trans-urocanate, kynurenate, cysteine, spermidine, cysteinyl glycine ↓ Decanoyl carnitine, 2-hydroxyheptanoate, 4-hydroxynonenal, glycerophosphoethanolamine, palmitoyl-linoleoyl-glycerol, stearoyl-linoleoyl-glycerol, cholate, n-acetylglutamine, pyroglutamine, tryptophan, cysteine s-sulfate
[108]	Bone Injuries	LC-MS/MS	Human bone marrow plasma	↑ Kynurenine

POP = postmenopausal osteoporosis; DOP = diabetic osteoporosis; RA = rheumatoid arthritis; OA = osteoarthritis; OS = osteosarcoma.

## 4. Metabolomics for Development of Bone Disease Treatment Methods

Metabolite measurements can provide valuable information to evaluate the response and progress of cells or organism to treatment methods. In the field of bone disease treatment development, currently, it has been employed in two main fields: osteoporosis therapeutics and biomaterials. 

### 4.1. Osteoporosis Therapeutics

Metabolomics can be applied to assess the therapeutic effect of drugs or dietary supplements on the recovery of an OP disorder. The effect of dietary Vitamin D on the bone metabolism of cats and dogs has been well reviewed [109]. The Icariin (IC) potential, a Chinese herbal bioactive molecule, in inducing bone differentiation has been proved previously [110,111]. It was reported that oral administration of IC to OP-induced 54-week-old hens as a supplement eventually leads to an increase in the BMD level of both femur and tibia by inducing changes in pyrimidine, taurine, and lipid metabolism. Furthermore, untargeted metabolomics of serum from hens treated with icariin helped identify eight altered metabolites: uridine, taurine, palmitic acid, adrenic acid, 30 fexofenadine, lysoPC, lysoPE, and 3-acetyl-11-keto-beta-boswellic acid, which can be considered markers for the early diagnosis of OP [112]. Similarly, yak bone collagen peptides (YBPs) showed the ability to induce bone differentiation in vitro. The enhanced effect of the intragastric administration of YBP on bone recovery in OVX rats was reported. The UPLC/Q-TOF-MS metabolomics of post-treated serum identified 41 potential biomarkers, mostly unsaturated fatty acids, the positive effects of which on postmenopausal OP have been proven. Indicatively, YBP supplementation demonstrated an increased DHA, arachidonic acid, taurine, citrulline, and a reduced serotonin metabolite concentration [113]. Osthole (OS) is the main bioactive integrant of Cnidium, which exhibited excellent treatment potential for OP. The ability of osthole to be introduced as an effective drug for postmenopausal OP (POP) was investigated. To this end, UPLC-Q-TOF/MS revealed that OS treatment leads to POP recovery by the regulation of 19 of the 28 highlighted metabolites related to the OVX-OP model (listed in Table 1). Osthole treatments revealed metabolic perturbations in amino acid, lipid, carbohydrate, bile acid, purine metabolism, and TCA cycle [102]. Bone-protective effects of lignin-rich fraction (SWR) as a Chinese herbal medicine were also reported. Serum LC/MS metabolomics of OVX rats was performed following 12 weeks post-treatment of SWR. The results proved that SWR administration leads to bone recovery by restoring the levels of 26 metabolites corresponding to estrogen deficiency which are involved in lipids, amino acids, tryptophan metabolisms, and anti-oxidative systems. In particular, upon SWR treatment, upregulation of superoxide dismutase and catalase was observed while serotonin was downregulated [70,114]. Additionally, metabolomics can provide valuable data to determine bone tissue treatment a gents’ side effect. A serum metabolomics study was performed to evaluate the side effects of long-term dexamethasone therapy in rats, and abnormal bone metabolism along with weight loss was observed, which was consistent with the reduced total ALP of serum. In detail, the dexamethason-treated rats exhibited six-fold downregulation of phenylalanine, lysine, and arginine and an increased tyrosine, hydroxyproline, and protein catabolism [115].

### 4.2. Biomaterials

Biomaterials are frequently used in surgical remediation of bone defects, especially for critical defects caused by traumatic fractures and bone tumor resection. Cell–material interactions play a critical role after the implantation of orthopaedic biomaterials. In recent years, few studies have demonstrated that metabolomics is a promising strategy to study that interaction and evaluate the implanted materials’ performance.

Poly-L-lactic acid (PLLA) has been used as implantable fracture fixation devices (bone screws and plates) for decades [116]. To study the role of PLLA in osteanagenesis, Araújo et al. employed NMR-based metabolomics to assess changes in metabolic processes in osteoblasts cultured on PLLA [117]. The decrease in glucose and triglycerides suggested that the energy metabolism was upregulated upon the interaction of PLLA. Furthermore, an increased lactate level followed by polymer breakdown can decrease the amount of intracellular radical oxygen species, reducing the intracellular redox state [6]. Though biocompatible, with its long degradation period, chronic inflammation is still observed with PLLA with the appearance of pro-inflammation cytokines (IL-1β, TNF and MCP-1) [118,119], even though the metabolism study of this chronic inflammation is still lacking.

Alginate hydrogel, a polysaccharide hydrophilic polymer with high water content, is another material that has been widely studied in biomedicine. As a highly tailorable polymer, the studies on alginate hydrogel scaffolds are usually performed in the context of the changed material composition by incorporating additives such as ions, acids, and peptides [120]. Oxidized alginate hydrogel functionalized by glycine-histidine-lysine, a peptide fragment of osteonectin [121], was developed by Klontzas et al. [122], and the osteogenesis properties were studied employing metabolome analysis. The reduction of lipid precursors, TCA cycle intermediates, and amino acid pools were observed in MSCs cultured with the modified hydrogel. Physical properties such as topography and stiffness of the material surfaces can also impact cell metabolism. Micro/nano surface structure has been an attractive topic in tissue engineering [6]. Researchers from two studies, conducted on titanium and polydimethylsiloxane, respectively, explored how the material surfaces impacted the metabolome with pillars in a pike shape [123,124].

Enhanced mitochondrial activity and lower ATP levels were identified in adherent cells, suggesting that macro pillars cause extra energy costs. The possible reason could be the cytophagy of the spires; however, further studies are needed to elucidate the underlying mechanisms. Additionally, material stiffness plays a role in adjusting cell behavior. Alakpa et al. synthesized hydrogels with different stiffness and studied the metabolome of cultured MSCs [125]. The steroid biosynthesis was enhanced in MSCs on hydrogel with a higher Young’s modulus (higher stiffness), while elevated glycerolipid biosynthesis level was enhanced on the lower Young’s modulus one. These findings indicate that MSCs tend to be osteogenically differentiated on rigid surfaces and chondrogenic differentiated on elastic or soft ones. Mechanical signals can also alter cell metabolism, as shown by Villaseñor et al. Metabolite analysis of cell medium samples obtained from mechanically stimulated osteocytes revealed a high citrate excretion and a decreased level of aspartate [126]. These alternations pointed to increased activity of the TCA cycle.

## 5. Conclusions and Outlook

Despite the overall maturity and broad application of metabolomics, it is still an emerging technique in bone research. However, it has become apparent that metabolism has a crucial role in bone homeostasis and disease at cellular and tissue levels. The metabolites profiling and metabolism pathway analysis can be combined with gene analysis, enzymatic activity assay, and biomaterial engineering to understand cell behavior at the molecular level. Metabolomics clarifies molecular mechanisms that are involved in the process of stem cell differentiation into bone-specific cells. This opens therapeutic possibilities to use metabolites directly or activating them indirectly, helping small molecules accelerate in vitro differentiation into bone cells and prevent unwanted differentiation. Despite the excellent understanding of the metabolic landscape of individual cell types involved in bone homeostasis, the interplay between these cells is currently understudied. It is apparent that cell–cell interactions have a crucial role, and future metabolomics studies are needed in this area. This could lead to new findings of metabolite signaling functions and the role of the metabolic microenvironment on cell functions.

Metabolomics has been successfully used to study disease-induced metabolic changes in vitro and in vivo and identify the potential biomarkers. Awareness of metabolic changes in the patient can lead to early diagnosis of bone-related diseases. Metabolomics can also accommodate a design of appropriate drugs to control the progress of the disease by following the decrease or increase in an effective biomarker in a disease. Additionally, as demonstrated by multiple animal model studies, metabolomics is a valuable tool to predict the therapeutic effect of a bone-protective agent on the recovery of bone diseases or their side effect on the normal function of bone. So far, several drugs have been designed to improve bone function, whereas their effectiveness can be challenged in further metabolomics studies. Furthermore, future metabolomics studies could be helpful to pinpoint the molecular targets for therapeutic agents.

The development and evaluation of biomaterials for bone regeneration is a fascinating and novel application area for metabolomics. As demonstrated in the available literature, biomaterial cues such as ions, oxygen, and regulatory metabolites affect cell metabolism and may cause changes in metabolic pathways and regeneration scenarios. The metabolic landscape of biomaterial–cell interactions appears to be uncharted territory. Although plenty of studies have been conducted on how biomaterials’ physiochemical properties can impact different cell behaviors, few of them were combined with metabolites profiling or metabolomics analysis. A combination of metabolomics and osteogenesis confirmation assays such as bone marker expression via a polymerase chain reaction, immunohistochemistry, and bone matrix mineralization could be a robust tool to guide the development and evaluate the performance of engineered biomaterials with desirable properties.

Altogether, metabolomics in the bone study is a promising area for interdisciplinary researchers, including material engineers, biologists, and clinicians.
